# A Peculiar Distribution of the Emerging Nematode *Angiostrongylus cantonensis* in the Canary Islands (Spain): Recent Introduction or Isolation Effect?

**DOI:** 10.3390/ani11051267

**Published:** 2021-04-28

**Authors:** Natalia Martín-Carrillo, Carlos Feliu, Néstor Abreu-Acosta, Elena Izquierdo-Rodriguez, Roberto Dorta-Guerra, Jordi Miquel, Estefanía Abreu-Yanes, Aarón Martin-Alonso, Katherine García-Livia, María Antonieta Quispe-Ricalde, Jordi Serra-Cobo, Basilio Valladares, Pilar Foronda

**Affiliations:** 1 Instituto Universitario de Enfermedades Tropicales y Salud Pública de Canarias, Universidad de La Laguna, Avda. Astrofísico F. Sánchez s/n, 38203 La Laguna, Canary Islands, Spain; nataliamartincarrillo@gmail.com (N.M.-C.); elenaizquierdorod@gmail.com (E.I.-R.); rodorta@ull.edu.es (R.D.-G.); esabya@gmail.com (E.A.-Y.); amalonso@ull.edu.es (A.M.-A.); kathegl16@gmail.com (K.G.-L.); bvallada@ull.es (B.V.); 2 Department Obstetricia y Ginecología, Pediatría, Medicina Preventiva y Salud Pública, Toxicología, Medicina Legal y Forense y Parasitología, Universidad de La Laguna, Avda. Astrofísico F. Sánchez s/n, 38203 La Laguna, Canary Islands, Spain; 3 Department of Biology, Health and Environment, Faculty of Pharmacy and Food Sciences, University of Barcelona, Av. Joan XXIII s/n, 08028 Barcelona, Catalunya, Spain; cfeliu@ub.edu (C.F.); jordimiquel@ub.edu (J.M.); 4 Faculty of Biology, IRBio (Research Institute of Biodiversity), University of Barcelona, Av Diagonal, 645, 08028 Barcelona, Catalunya, Spain; serracobo@areambiental.com; 5 Nertalab S.L. José Rodríguez Mouré, 4, bajo, 38008 Santa Cruz de Tenerife, Canary Islands, Spain; gerencia@nertalab.es; 6 Department Matemáticas, Estadística e IO, Faculty of Science, Universidad de La Laguna, 38206 La Laguna, Canary Islands, Spain; 7 Department Biología, Faculty of Science, Universidad Nacional San Antonio Abad de Cusco, Cusco 08000, Peru; maquispe@hotmail.com; 8 Department Biología Evolutiva, Ecología y Ciencias Ambientales, Facultad de Biología, Universidad de Barcelona, Av. Diagonal, 645, 08028 Barcelona, Catalunya, Spain

**Keywords:** *Angiostrongylus cantonensis*, Canary Islands, emerging disease, rat lungworm, *Rattus*, expanding parasite, eosinophilic meningitis

## Abstract

**Simple Summary:**

*Angiostrongylus cantonensis*, commonly known as the rat lungworm, is considered the leading cause of eosinophilic meningitis in humans. It is an emerging zoonotic parasite, endemic to the temperate and tropical zones of the Far East, Southeast Asia and the Pacific Islands, that has expanded to all continents with the exception of Antarctica. Considering the recent finding of this parasite in rats from the Canary Islands, the aim of this study was to determine its current distribution in these islands in order to highlight the risk sources for angiostrongyliasis in the archipelago. We also analyzed the environmental conditions that could determine distribution. *A. cantonensis* was detected in only one of the eight islands that constitute this archipelago, i.e., in the north part of the island, which presents better environmental conditions than the south for the parasite to establish itself. This limited distribution could indicate a recent introduction of the parasite in the Canaries or an isolation effect that has not allowed the expansion to the other islands. The presence of *A. cantonensis* implies risks for humans and other animals that justify the need of control measures to prevent the expansion to other similar areas of the archipelago.

**Abstract:**

*Angiostrongylus cantonensis* is an emerging zoonotic nematode recognized as the leading cause of eosinophilic meningitis in the word. After its discovery in China, it was recorded in 30 countries worldwide. Recently, it has expanded to new areas such as South America and it has been recently found in the Atlantic island of Tenerife (Canary Islands). In order to characterize the distribution of *A. cantonensis* in the Canary Islands, the lungs of 1462 rodents were sampled in eight islands of the archipelago over 13 years and were then analyzed for *A. cantonensis*. Remarkably, the parasite was detected only in Tenerife, in *Rattus rattus* (19.7%) and *Rattus norvegicus* (7.14%). They were concretely in the northern part of the island, which had a warmer and more humid climate than the south and main cities. The absence of this nematode in other islands with similar environmental conditions could be explained by an isolation effect or by a recent introduction of the parasite in the islands. Besides, the presence in Tenerife of the most invasive lineage of *A. cantonensis* reinforced the hypothesis of a recent introduction on this island. This study highlights the need to implement control measures to prevent the expansion to other areas in order to avoid the transmission to humans and other animals.

## 1. Introduction

*Angiostrongylus cantonensis*, the rat lungworm, is a zoonotic nematode belonging to the superfamily Metastrongyloidea (family Angiostrongylidae) [1]. It was originally identified in the Guanghou region in China in the brown rat, *Rattus norvegicus* [2]. Traditionally, *A. cantonensis* is endemic to the temperate and tropical parts of the Far East, Southeast Asia, and Pacific Islands [1]. The parasite has also been reported in Africa, the Caribbean islands (i.e., Puerto Rico, Dominican Republic, Cuba, and Jamaica), Australia, and Hawaii [1]. Reports of newly invaded territories keep occurring—e.g., the Canary Islands (Spain) (located in the Northwest African margin [3], Mallorca (Spain) [4], Uganda [5], and the southern part of the United States (i.e., Oklahoma and Florida) [6,7]. Therefore, it has reached all continents except Antarctica, suggesting an expansion of the parasite.

The life cycle of this parasite involves rats as the definitive host, mollusks as intermediate hosts, and crustaceans, raptors, planarians, frogs, and lizards, among others, as paratenic hosts [8]. Rats are infected with *A. cantonensis* after ingesting third-stage larvae. The larvae migrate to the central nervous system, where they become fourth- and fifth-stage larvae via two molts. Finally, they turn into adult worms [9]. Humans, companion animals, and wildlife are accidental dead-end hosts for *A. cantonensis* [10]. It is known that human infections are usually acquired by purposeful or accidental ingestion of infective larvae in terrestrial mollusks, planarians, and freshwater crustacea or vegetables containing the infective larvae [11]. In the case of humans, once swallowed, the infective larvae are digested from those vectors and invade the intestinal tissue, causing human enteritis, before passing through the liver. Cough, rhinorrhea, sore throat, malaise, and fever can develop when the worms move through the lungs. Finally, the larvae reach the central nervous system in about two weeks, causing eosinophilic meningitis and eosinophilic pleocytosis [12], or they move to the eye chamber causing ocular angiostrongyliasis [13]. A wide variety of animal species have been identified as accidental hosts for *A. cantonensis*, such as dogs, horses, brush-tailed possums, Bennett’s kangaroos, rufous bettongs, black and gray-headed flying foxes, or tawny frogmouths [14]. Neurological abnormalities have been recorded in some of these wildlife and domestic animals [15]. This wide range of species may indicate that the distribution of this emerging parasite continues to expand, posing a possible risk to humans.

The first human case of angiostrongyliasis was reported in Taiwan, where it was identified as a human pathogen in 1945 [16]. Now, it is recognized as the leading cause of eosinophilic meningitis worldwide [17]. Over 3500 cases of human angiostrongyliasis were recorded from more than 30 countries in 2017 [18] (i.e., USA, Australia, New Caledonia, Philippines, Cuba, Ecuador, Puerto Rico, Brazil, Egypt, Nigeria, and Madagascar, among other countries [6,19,20]).

One of the theories for the appearance of this nematode outside of Southeast Asia is that its spread has been facilitated by the introduction of the giant African snail, *Achatina fulica*, which could have carried the parasite across the Pacific where rat populations were already established [21,22,23]. Hochberg et al. (2007) [24] proposed that the spread of *A. cantonensis* has been driven by climate change factors and globalization, as well as by the increasing international trade. Moreover, many authors attribute the spread of *A. cantonensis* to sheer diversity of its intermediate hosts [25] and high adaptability to a new intermediate host species. Rats were and will continue to be the principal agents of expanding the parasite beyond the Indo-Pacific area [11].

**Figure 1:**
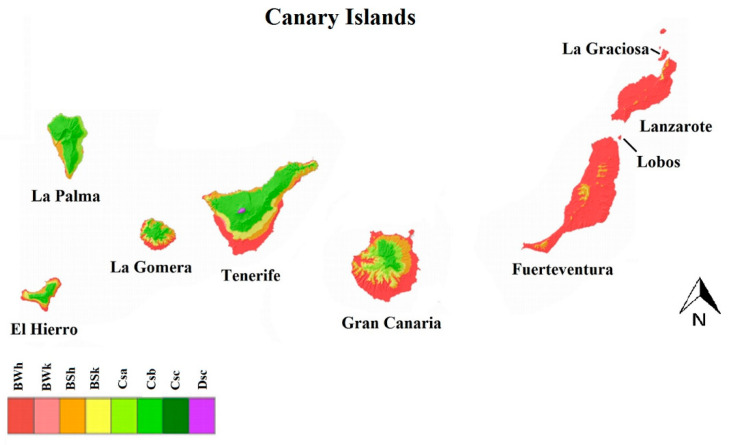
Köppen–Geiger climate classification of the Canary Islands, modified from State Meteorological Agency of Spain [26]. BWh (hot desert), BWk (cold desert), BSh (hot steppe), BSk (cold steppe), Csa (temperate with hot and dry summer), Csb (temperate with dry and warm summers), CSc (temperate with dry and cool summers), and DSc (cold without a dry season and a fresh summer).

The Canary Archipelago is composed of eight volcanic islands and five islets. Tenerife and Gran Canaria are the central and most populated islands of the archipelago and the capitals of the two provinces, respectively. They are part of the Macaronesian region located on the NW African coast (13°23′–18°8′ W and 27°37′–29°24′ N). Each island has particular geographic and climatic characteristics. Overall, the climate is subtropical moderated by the oceanic Canary Current, prevailing northeast trade winds, and especially by altitude. According to the Köppen-Geiger climate classification [26], the Canary Islands present hot desert (BWh) or cold desert (BWk) climates, characterized by evaporation that exceeds precipitation on average but is less than half the potential evaporation, with an average temperature of >18 °C (BWh) and <18 °C (BWk). Hot steppe (BSh) and cold steppe (BSk) climates are characterized by evaporation that exceeds precipitation on average but is less than the potential evaporation; the difference between these two measures is less than what has been found in a BW climate. The average temperature is >18 °C (BSh) and <18 °C (BSk). The temperate climate with hot and dry summers (Csa) is characterized by hot summers with an average temperature of >22 °C in the warmest month. The temperate climate with dry and warm summers (Csb) is characterized by warm summers with an average temperature in the hottest month of ≤22 °C, along with four months or more of average temperatures of >10 °C. The distribution of these climates in the different islands of the archipelago is represented in Figure 1.

With the presence of these climatic differences and the influence of the oceanic Canary Current, the prevailing northeast trade winds, altitude, and up to six zonal ecosystems have been described. From a lower to higher altitude, there is the coastal scrub (0–300 m), thermophilic forest (300–500 m), Monteverde (composed by Fayal-Brezal and Laurel forest) (500–1200 m), pine forest (1200–2000 m), summit scrub (>2000 m), and peak vegetation (>2700 m) [27,28]. In the Canary Islands, *A. cantonensis* was described for the first time in 2010 in specimens of *R. rattus* from the island of Tenerife in the municipalities of San Cristóbal de La Laguna and Tegueste [3], as located in the thermophilic forest and Monteverde bioclimatic floors.

Given the expansion of this emerging parasite and the health implications for humans and wildlife, there is a need to determine the distribution of *A. cantonensis*. For this reason, the aims of this study were to document the current distribution of this parasite across the Canary Islands by screening islands and islets, and to determine the distribution in the island of Tenerife, where the nematode was found. We analyzed the different bioclimatic conditions that could determine its presence. The knowledge of the risk areas could be of useful for preventing and controlling the transmission and spread of the parasite.

## 2. Materials and Methods

**Figure 2:**
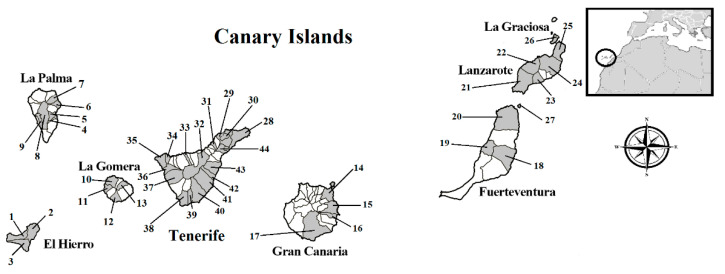
Map of the rodent sampling locations in the Canary Islands. The municipalities analyzed are shaded in gray. 1: Frontera, 2: Valverde, 3: El Pinar, 4: Breña Baja, 5: Breña Alta, 6: Puntallana, 7: San Andrés y Sauces, 8: El Paso, 9: Los Llanos de Aridane, 10: Vallehermoso, 11: Valle Gran Rey, 12: Alajeró, 13: Hermigua, 14: Las Palmas de Gran Canaria, 15: Telde, 16: Ingenio, 17: San Bartolomé de Tirajana, 18: Antigua, 19: Betancuria, 20: La Oliva, 21: Yaiza, 22: Tinajo, 23: Tías, 24: Teguise, 25: Haría, 26: La Graciosa, 27: Islote de Lobos, 28: Santa Cruz de Tenerife, 29: San Cristóbal de La Laguna, 30: Tegueste, 31: El Sauzal, 32: La Orotava, 33: La Guancha, 34: El Tanque, 35: Buenavista, 36: Santiago del Teide, 37: Guía de Isora, 38: Arona, 39: San Miguel de Abona, 40: Granadilla, 41: Arico, 42: Fasnia, 43: Güímar, 44: El Rosario.

In a period of 13 years (between 2007 to 2020), a total of 1462 rodents belonging to the species *R. rattus*, *R. norvegicus,* and *Mus musculus domesticus* were captured from the Canary Islands: 331 were from El Hierro (87 *R. rattus* and 244 *M. m. domesticus*); 117 were from La Palma (30 *R. rattus*, 2 *R. norvegicus*, and 85 *M. m. domesticus*); 49 were from La Gomera (16 *R. rattus* and 33 *M. m. domesticus*); 478 were from Tenerife (283 *R. rattus*, 14 *R. norvegicus*, and 181 *M. m. domesticus*); 61 were from Gran Canaria (19 *R. rattus*, 1 *R. norvegicus*, and 41 *M. m. domesticus*); 131 were from Fuerteventura (25 *R. rattus* and 106 *M. m. domesticus*); 237 were from Lanzarote (33 *R. rattus* and 204 *M. m. domesticus*); 37 were from La Graciosa (37 *M. m. domesticus*); and 21 were from the islet of Lobos (21 *M. m. domesticus*) (Figure 2).

Tenerife is divided into a total of 31 municipalities, distributed between the north, south, and metropolitan areas. The traps were set in intra-, peri-, and extra-domiciliary settings, corresponding to inside, around, and far from inhabited areas, belonging to urban and rural areas, on different bioclimates floors representing a wide range of ecosystems. The rural areas mainly included arid habitats, crop fields, private gardens and farms, laurel forest, and fayal-brezal. Urban areas, however, corresponded to highly populated zones of the two main cities of Tenerife. Unlike the other islands included in this study, the low altitude of Lanzarote, La Graciosa, Fuerteventura, and Lobos isolate gave rise to little contrast in the vegetation, and traps were set in only one biotope with crop fields. More intensive trapping was carried out in laurel forests, since *A. cantonensis* was recovered in this ecosystem for the first time in the archipelago.

Animal work was approved in accordance with the Spanish Government Laws 42/2007 and RD 630/2013, the Canary Government law 151/2001 (expedient references FYF141/10, FYF205/09, EEI-007/2019, ADL/mjb, MRR/rsh, A/EST-030/2016, AFF115/16, and EEI-007/2019), and the Ethic Committees of Research and Animal Wellness of Universidad de La Laguna (Protocol number CEIBA2018-0330). Captured rodents were euthanized in either a CO_2_ chamber (rats) or via cervical dislocation (mice). Afterwards, they were sexed, weighed, and necropsied. The lungs were extracted and individually placed in petri dishes containing phosphate-buffered saline and observed under a stereomicroscope to search the adult forms of *A. cantonensis*. Some of the nematodes obtained from the parasitized rodents were stored individually in 1.5 mL tubes with 70° alcohol for morphological study, whereas the others were placed individually in 1.5 mL tubes containing absolute alcohol and refrigerated at 4 °C or stored without any preservative at −80 °C for future molecular analyses. The nematodes were clarified with Amman lactophenol and studied morphologically with the use of a light microscope. Morphological identification of the adult nematodes was based on Chen (1935), Kinsella (1971), Drozdz et al. (1975), and Ubelaker (1986) [2,29,30,31].

The mean intensity of *A. cantonensis* in rats was calculated by dividing the total number of nematodes obtained by the number of infected rats. Data analyses was carried out using the R 3.5.1 [32] statistical software. The prevalence is presented as proportions for categorical data. Moreover, 95% Clopper Pearson confidence intervals (95% CI) were evaluated using the approximate or exact method, as appropriate. The numbers of parasites for male and female rodents were non-normally distributed, as assessed by Shapiro-Wilk test and visual histogram inspections. Hence, a Mann-Whitney U test was run to determine if there were differences in the number of parasites between male and female rodents. A chi-square test, used for association, was conducted between the presence of *A. cantonensis* and the capture area of rodents, in both the north or south of Tenerife island. All expected cell frequencies were greater than five.

The prevalence of *A. cantonensis* on the island of Tenerife was represented in a heat map using the inverse distance weighting (IDW) interpolation method integrated in the interpolation complement of the quantum GIS software (QGIS, Open-Source Geospatial Foundation Project, Hannover, Germany), version 3.16.

## 3. Results

*A. cantonensis* was found only in Tenerife island, with a general prevalence of 21.20% (57/297; 95% CI:14.87–24.14). Further, 19.7% (56/283; 95% CI: 15.31–24.91) was found for *R. rattus* and 7.14% (1/14; 95% CI: 0.18–33.87) was found for *R. norvegicus.* None of the *M. m. domesticus* was infected.

**Figure 3:**
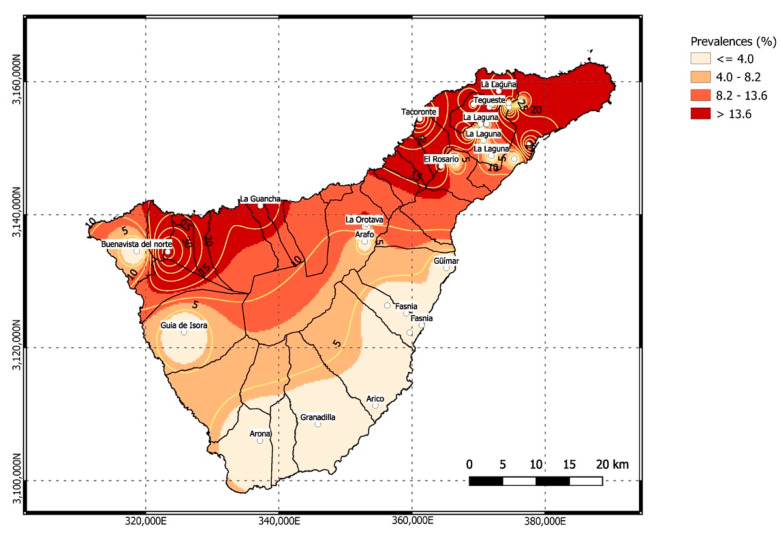
Heat map of the prevalence of *Angiostrongylus cantonensis* in the island of Tenerife, using the Inverse Distance Weighting (IDW) interpolation method integrated in the interpolation complement of the Quantum GIS software (QGIS).

Prevalence estimates obtained in the different sampled areas of this island are shown in Table 1. All the positive rats were located in the north, i.e., El Rosario (21.05%), El Sauzal (100%), El Tanque (37.5%), La Guancha (16.6%), La Orotava (20%), and Tegueste (11.9%) municipalities, as well as in the two main cities of Tenerife, Santa Cruz de Tenerife (33.33%) and San Cristóbal de La Laguna (22.82%). No positive rats were found in the other study areas (Figure 3). The overall mean intensity of *A. cantonensis* in the rats was 8.37 (477/57), ranging from 1 to 60 individuals being 5.25 (21/4; 1 to 10) from El Rosario, 6.25 (24/4; 5 to 10) from El Sauzal, 3.83 (23/6; 1 to 5) from El Tanque, 1 (1/1; 1) from La Guancha, 9.57 (67/7; 1 to 26) from La Orotava, 6.4 (128/20; 1 to 20) from San Cristóbal de La Laguna, 11.25 (45/4; 5 to 20) from Santa Cruz de Tenerife, and 16.7 (167/10; 1 to 30) from Tegueste. There was not a statistically significantly difference in the number of parasites between male and female rodents (*p* = 0.918). However, there was a statistically significant association between the presence of *A. cantonensis* and the north and south capture areas of rodents (*p* = 0.002).

The biotopes where *A. cantonensis* was detected corresponded to Monteverde, thermophilic forest, cultivated fields, and open fields. The rats with *A. cantonensis* were obtained in extra-, peri- and intra-domiciliary areas. In the two main cities of Tenerife, the parasite was found in the center of the cities, close to the heavily populated areas. In the case of Santa Cruz de Tenerife, it was detected in the wet areas of ravines.

## 4. Discussion

The rat populations of the Canary Islands are composed by two species: *R. rattus* and *R. norvegicus*, which have both been introduced in the archipelago and are distributed throughout the islands [33]. As they are definitive hosts for *A. cantonensis* [12], the high density and wide distribution of these rodent species in the Canary Islands [33] highlight the importance of this study from a public health point of view, since rats are necessary for establishing of *A. cantonensis* in new locations [1].

It is important to note the high biodiversity of gastropods species (snails and/or slugs) present in the Canaries [34]. Three of them, two of which are endemics to the Canary Islands, have demonstrated their capacity as intermediate hosts of *A. cantonensis*, with a general prevalence for *A. cantonensis* of 19.3% of the mollusks studied. Therefore, other mollusk species should be analyzed in order to determine their potential as intermediate hosts of *A. cantonensis* [35]. *A. cantonensis* could have been introduced to the Canary Islands as a result of the unintended importation of definitive rodent hosts (as commented by Wang et al. [1]) or the importation of intermediate gastropods species on ships and airplanes.

Inter- and intraspecific genetic variations in the genus *Angiostrongylus* have been reported using partial sequencing of the cytochrome oxidase subunit I (COI) gene [36] because the *ac8* haplotype is considered to be the most widespread and invasive [37]. Červená et al. [38], using next-generation whole-genome sequencing methods, assembled and analyzed the complete mtDNA from four geographically disparate localities, including Tenerife, of *A. cantonensis* outside the endemic range of the parasite. The observed uniformity of the invasive strains implies that only certain genetic lineages of *A. cantonensis* have the ability to become globally invasive because *ac8* haplotype has the most widespread lineage, and is the most similar to the haplotype found in Tenerife.

*A. cantonensis* was found only in Tenerife, specifically in its northern locations that have higher precipitation and the presence of horizontal rain biotopes. Nevertheless, it is curious that *A. cantonensis* has not been found in La Palma, El Hierro, or La Gomera, since these islands present the same biotopes as Tenerife. Further, these locations have the necessary factors for the settlement of *A. cantonensis*. In Gran Canaria, there is some representation of laurel forest, but it is highly degraded compared to the other islands. These biotopes also present warmer temperature, which supports the parasite in a general tropical and subtropical distribution range [25]. This is related to the threshold temperature for larval development in its intermediate mollusk hosts [39], as well as its susceptibility to dry conditions. Unlike the other islands and islets included in this study, Fuerteventura, Lanzarote, La Graciosa, and Lobos have low altitude that gives rise to little contrast in their vegetation, in addition to warmer temperatures and less frequent rains.

On the other hand, it is worth noting the presence of *A. cantonensis* in the city of Santa Cruz de Tenerife, the capital of Tenerife, where the main commercial port of the island is located and where the transit of ships and people is constant. However, it is curious that, in the island of Gran Canaria, this nematode was not found. The ports of the Canary Islands have been an important enclave in support of Atlantic navigation since colonial expansion, acting as a connection between European, African, and American ports. Thanks to the economic process of globalization, transporting goods is increasingly important in order to sustain consumption levels in today society. Maritime trade accounts for 80% of the total transport of goods carried out across the world. In 2019, the port of Santa Cruz de Tenerife registered a port traffic of 1,664,824 ships [40], while the port of Las Palmas de Gran Canaria had a traffic of 1,514,765 ships [41]. The location of both ports played an essential role in establishing *A. cantonensis*, since the environment differs between them. The port of Tenerife is located in Santa Cruz de Tenerife, where the parasite has been to shown to have adequate conditions for settlement, while the port of Gran Canaria is located in Las Palmas de Gran Canaria, with a more urbanized environment and arid climate.

In the ports of the rest of the studied islands, the transit of cargo ships or passenger transport has been much lower over the years than in these two main ports of Tenerife and Gran Canaria. Therefore, this may be one of the causes why *A. cantonensis* has not yet entered these islands. Taking into account all of these factors, the observed distribution of *A. cantonensis* in only one of the Canary Islands can be explained by a recent introduction of this parasite in the archipelago or it can be explained by the geographic isolation of island ecosystems.

Regarding the environmental conditions for the development of this zoonotic pathogen, some bioclimatic variables significantly contribute to predicting the potential distribution of the parasite, such as temperature and precipitation, because they have a significant impact on pathogens, vectors, and reservoirs [42]. Specifically, in Tenerife, an irregular distribution was observed, which was probably due to environmental conditions, since the areas where it was found coincide with areas of trade winds incidence and horizontal rain. For this reason, we expect this parasite to spread to similar areas both in the Canary Islands and beyond. Determining how environmental factors may affect the distribution of *A. cantonensis* will allow a better forecast of its potential spread and will facilitate a more targeted intervention to avoid public health risks. Various studies suggest the effects of global climate change increases balanced habitat suitability in some areas and decreased ones elsewhere [42].

As in most natural systems, climate change affects hosts and parasites, altering survivorship, reproduction, and transmission, among other factors. In addition, environmental and socioeconomic systems are rapidly changing, modifying interactions among humans, animals, and their pathogens [43,44]. According to Lv et al. [39], the sum of environmental and biological factors may determine the critical conditions required for the parasite and its intermediate hosts to survive. The effects of global climate change and globalization are hypothesized to influence disease range expansions (via pathogen spread) and indirect expansions (via reservoirs, hosts, or vector range expansions). This will increase the frequency of disease outbreaks and expand the pool of at-risk populations. The global model for the present distribution of *A. cantonensis* predicts that the most suitable habitat is located near the equator in tropical to subtropical regions [25].

All the same, the positive association between temperature and pathogen transmission can be offset by the total bioclimatic survival requirements of a pathogen. If these requirements are not met, the mortality of the host, the vector, and/or the pathogen will increase, and the pathogen will not settle in areas where it is predicted to settle [45]. As numerous studies have shown over the years, since this zoonotic pathogen was discovered, its presence and risk as an emerging disease have not stopped growing because its distribution is no longer limited to only endemic regions [18]. In conclusion, it is worth highlighting the importance of controlling the spread of *A. cantonensis* to areas where it is not currently present.

Preventing the spread of intermediate and definitive host for *A. cantonensis* must be a principal goal of authorities in those countries where the presence of this parasite has been found. In order to avoid the spread of this emerging parasite, control measures must be adopted into the transport of goods in ports and airports. In China, efforts to prevent its further spread and population expansion includes the use of chemicals and biological control agents to control the snails [46].

Although raw snails are generally not part of the main diet of people on the Canary Islands, some species such as *Cornu aspersum* are used in regions of the Canary Archipelago for the preparation of typical dishes. Yet there is a risk associated with ingestion if they are undercooked [47]. In order to avoid the transmission of infective larvae by infected invertebrates or vegetables contaminated by mollusks infected with this parasite, which have not been properly washed for consumption, health education, rigorous food inspection, and surveillance are all needed to prevent potential angiostrongyliasis outbreaks. It is also important to note the need to carry out studies in humans because some cases of eosinophilic meningitis caused by infection with *A. cantonensis* may not be reported by a faulty diagnosis, as it can occur in the veterinary sector with companion animals, such as dogs [10].

## 5. Conclusions

The presence of *A. cantonensis* in the Canary Islands was found to be limited to the northern part of the island of Tenerife, where incidence of the oceanic Canary Current and trade winds are more prominent than in the south or main cities. The introduction of the nematode could be caused by globalization, including the role of the Canary Islands as a maritime connection between European, African, and American ports, with the entrance by ships in Tenerife as one of the most important ports. Their absence in Gran Canaria, the other most important port, could be due to unsuitable environmental conditions for its establishment. A recent introduction of *A. cantonensis* in the archipelago could explain the presence in one of the islands, since there were others with similar environmental conditions. On the other hand, the isolation effect could play a role in this peculiar distribution. From a public health point of view, the presence of this emerging parasite in highly populated areas in Tenerife implies a risk of transmission to humans. Control measures must be taken to prevent the spread of this parasite to other areas with similar environmental conditions in order to prevent angiostrongyliasis.

## Figures and Tables

**Table 1:** Prevalence of *Angiostrongylus cantonensis* in the island of Tenerife, divided by rodent species as well as location.

Locations	*Rattus rattus*	*Rattus norvegicus*	*Mus musculus domesticus*	Total
	P (%) (+/n)	P (%) (+/n)	P (%) (+/n)	P (%) (+/n)
**North**	**22.79 (31/136)**	**9.09 (1/11)**	**0 (0/107)**	**12.59 (32/254)**
Buenavista del Norte	0 (0/5)	0 (0/1)	0 (0/2)	0 (0/8)
El Rosario	21.05 (4/19)	0 (0/0)	0 (0/4)	17.39 (4/23)
El Sauzal	100 (4/4)	0 (0/9)	0 (0/9)	18.18 (4/22)
El Tanque	37.5 (6/16)	0 (0/0)	0 (0/1)	35.29 (6/17)
La Guancha	16.6 (1/6)	0 (0/0)	0 (0/5)	9.09 (1/11)
La Orotava	20 (7/35)	0 (0/0)	0 (0/54)	7.86 (7/89)
Tegueste	17.64 (9/51)	100 (1/1)	0 (0/32)	11.90 (10/84)
**South**	**0 (0/28)**	**0 (0/8)**	**0 (0/13)**	**0 (0/49)**
Arico	0 (0/0)	0 (0/6)	0 (0/0)	0 (0/6)
Arona	0 (0/2)	0 (0/0)	0 (0/2)	0 (0/4)
Fasnia	0 (0/19)	0 (0/2)	0 (0/1)	0 (0/22)
Granadilla	0 (0/1)	0 (0/0)	0 (0/3)	0 (0/4)
Guía de Isora	0 (0/6)	0 (0/0)	0 (0/0)	0 (0/6)
Güímar	0 (0/0)	0 (0/0)	0 (0/3)	0 (0/3)
San Miguel de Abona	0 (0/0)	0 (0/0)	0 (0/4)	0 (0/4)
Santiago del Teide	0 (0/0)	0 (0/0)	0 (0/1)	0 (0/1)
**Main cities**	**24.04 (25/104)**	**0 (0/0)**	**0 (0/51)**	**16.13 (25/155)**
San Cristóbal de La Laguna	22.82 (21/92)	0 (0/0)	0 (0/36)	16.4 (21/128)
Santa Cruz de Tenerife	33.33 (4/12)	0 (0/0)	0 (0/15)	14.81(4/27)
Total	21.20 (60/283)	7.14 (1/14)	(0/181)	12.76 (61/478)

P (%): prevalence of *Angiostrongylus cantonensis*; +/n: number of animals with *A. cantonensis*/number of animals analyzed. These prevalence’s have been calculated including the data from the study carried out by Foronda et al. [3] and the data taken up to the year 2020.

## Data Availability

The data presented in this study are available upon request from the corresponding author. The data is not publicly available due to internal laboratory policy.
